# Case Series of Three Patients with Disseminated Gonococcal Infection and Endocarditis

**DOI:** 10.5811/cpcem.2021.7.53404

**Published:** 2021-10-26

**Authors:** Phillip Moschella, Hannah Shull, Mark Pittman, Alex Gleason, Prerana Roth

**Affiliations:** * Prisma Health-Upstate, Department of Emergency Medicine, Greenville, South Carolina; † Prisma Health-Upstate, Department of Infectious Diseases, Greenville, South Carolina

**Keywords:** Neisseria gonorrhoeae, gonococcal endocarditis, disseminated gonococcal infection, case report, case series

## Abstract

**Introduction:**

The increasing incidence of *Neisseria gonorrhoeae* infections and emergence of cephalosporin-resistant strains means the threat of disseminated gonococcal infection and endocarditis needs to be reimagined into the differential diagnosis for patients treated in the emergency department (ED) for sexually transmitted infections and for endocarditis itself. Only 70 cases of disseminated gonococcal infection (DGI) with endocarditis had been reported through 2014.^1^–^4^ In 2019, however, an outbreak of DGI with one case of endocarditis was reported.^5^ This case series of three patients with DGI and endocarditis, in addition to the recent outbreak, may represent a warning sign for reemergence of this threat.

**Case Report:**

We describe three cases within a recent three-year period of gonococcal endocarditis as seen and treated at our institution. These cases show divergent presentations of this insidious disease with both classical and atypical features. One case displayed a classic migratory rash with positive urine testing and a remote history of sexually transmitted infections, while another patient developed isolated culture-confirmed endocarditis with negative cervical testing and imaging, and the final case was a male patient who presented to the ED with fulminant endocarditis as the first ED presentation of infection.

**Conclusion:**

Secondary to an overall rise in incidence and possibly due to increasing antibiotic-resistance patterns, gonococcal endocarditis should be included in the differential diagnosis of any case of endocarditis. Reciprocally, increased vigilance should surround the evaluation of any patient for sexually transmitted diseases while in the ED for both the development of DGI and endocarditis.

## INTRODUCTION

*Neisseria gonorrhoeae*, a Gram-negative diplococcus, primarily infects the mucous membranes of the urethra and cervix. Disseminated gonococcal infection (DGI) occurs in 0.5–3% of patients with development of endocarditis in up to 1–2% of DGI patients. Before 1938, *N. gonorrhoeae* was responsible for up to 26% of all bacterial endocarditis and was uniformly fatal. With the advent of effective antibiotic therapy, gonococcal endocarditis has become rare, yet mortality even with appropriate treatment remains high (19–20%).^1^–^3^ It is important to re-emphasize the pathogenic capacity of *N. gonorrhoeae given the following*: 1) the increasing overall incidence of gonorrhea infections in the United States (US); 2) the emergence of new treatment-resistant strains; and 3) the high mortality rate associated with gonococcal endocarditis*.* A recent literature review found only 70 cases of gonococcal endocarditis had been reported in the literature after 1939.^4^ Since then, a recent outbreak of DGI with one case of endocarditis was reported.^5^ This report describes three additional separate cases of gonococcal endocarditis as seen within our emergency departments (ED) between 2017–2020. These patient presentations represent several ways in which this insidious disease may present and how gonococcal endocarditis was diagnosed and successfully treated. The comparative rarity of reporting on gonococcal endocarditis prior to 2014 and the fact that our single institution has had three such patients within a three-year period should serve as a warning for increased awareness and surveillance in the ED.

## CASE SERIES

### Case 1

A 26-year-old female presented to the ED in 2017 with a chief complaint of several days of nausea, abdominal pain, and a newly painful right knee associated with a migratory erythematous rash. The patient stated that one day prior, the same rash and pain were present on her contralateral knee but resolved with no treatment. Her past medical history (PMH) was significant for bipolar disorder and a chlamydia infection, successfully treated the prior year. Her social history was significant for tobacco and alcohol use, and no reported drug use. Her triage vital signs were temperature 36.7^o^ Celsius, heart rate 87 beats per minute, blood pressure of 137/87 millimeters mercury (mm Hg), respiratory rate of 26 breaths per minute, and pulse oximetry of 99% on room air. Her physical exam (PE) was notable only for a large circular erythematous rash over the proximal aspect of her right knee and leg with full range of motion with minimal to no pain and no murmur on cardiac auscultation (Image).

She refused a vaginal exam. Significant laboratory results were as follows: white blood cell count 12.2 thousand per cubic millimeter (k/mm^3^) (reference range 4.5–11.5 K/ mm^3^; C-reactive protein 3.5 milligrams per liter (mg/L) (reference less than 3.0mg/L); erythrocyte sedimentation rate 16 mm per hour (hr) (0–20 mm/hr); procalcitonin less than 0.05 nanograms per milliliter (ng/mL) (less than 0.1 ng/mL); rapid plasmin reagent was negative; urinalysis showed greater than 182 white blood cells per high-power field (HPF); greater than 182 red blood cells per HPF, and 5 squamous epithelial cells per HPF; urine pregnancy test was negative; and urine gonorrhea polymerase chain reaction was positive. A computed tomography (CT) of the abdomen and pelvis with intravenous (IV) contrast showed cholelithiasis without acute cholecystitis. Given her prior history of chlamydia infection, the migratory rash and joint pain, DGI was presumed, and blood cultures were obtained with initiation of IV ceftriaxone. Arthrocentesis was deferred. Several additional labs were ordered to evaluate for autoimmune disorders upon admission. Throughout her admission, the patient manifested several fevers across numerous nursing shifts with several sets of blood cultures (four total sets including the set obtained upon admission) obtained. All eventually showed no growth. A transthoracic echocardiogram (TTE) was performed on hospital day four, which showed an abnormally thickened aortic valve. Transesophageal echocardiogram confirmed a mobile vegetation presumed to be gonococcal endocarditis, which was successfully treated with four weeks of IV ceftriaxone.

**CPC-EM Capsule**

What do we already know about this clinical entity?

*Gonococcal endocarditis following disseminated infection, although rare, has a high degree of mortality (~20%) even despite modern therapies.*

What makes this presentation of disease reportable?

*This case series highlights three separate cases in three years at one institution of this historically rare disease.*

What is the major learning point?

*The emergence of treatment resistant gonorrhea and increasing overall incidence may herald an unfortunate rise of gonococcal endocarditis.*

How might this improve emergency medicine practice?

*The differential for endocarditis should include gonorrhea with added vigilance during evaluation and treatment for emergency department patients with sexually transmitted infections.*

### Case 2

A 25-year-old female presented to the ED in 2018 with chief complaint of fever for one day and three weeks of cough, chest tightness, shortness of breath, abdominal pain, nausea, vomiting, fatigue, and an unintentional 10-pound weight loss. She had a PMH significant for mild asthma treated with a rescue inhaler and a remote history of chlamydia. She denied any alcohol, tobacco, or recreational drug use. Her triage vital signs were temperature 36.7^o^C, heart rate 87 beats per minute, blood pressure of 137/87 mm Hg, respiratory rate of 26 breaths per minute, and pulse oximetry of 99% on room air. Her PE was significant for a grade IV/VI systolic ejection murmur heard upon cardiac auscultation at the base of the heart. Significant laboratory results were as follows: white blood cell count 14.3 K/mm3; C-reactive protein 118.4 mg/L; erythrocyte sedimentation rate 83 mm/hr, procalcitonin 0.74 ng/mL, D-dimer 2.72 micrograms (μg)/mL (reference range <0.42 μg/mL), and a negative urine pregnancy. A CT angiogram of the chest was obtained and negative for pulmonary embolism but did show patchy infiltrates in the left lower lobe.

Blood cultures were drawn in the ED, and the patient was initially started on IV vancomycin and piperacillin-tazobactam and admitted to the hospital. A TTE was obtained upon admission and showed severe mitral regurgitation, concern for a ruptured chordae tendinea, and thickening/vegetations on the leaflets concerning for endocarditis. On hospital day two, both sets of blood cultures grew aerobic Gram-negative diplococci, which were identified as *N. gonorrhoeae* using a VITEK analyzer (*bioMérieux*, Inc. USA, Durham, NC). The isolate was sensitive to ceftriaxone. A pelvic exam found no discharge or cervical motion tenderness, and cervical gonococcal and chlamydia deoxyribonucleic acid probes were negative. A transvaginal ultrasound showed no abscess or signs of pelvic inflammatory disease. The patient underwent mitral valve replacement on hospital day five, and successfully completed six weeks of IV ceftriaxone.

### Case 3

A 20-year-old male presented to the ED in 2020 with a chief complaint of one month of worsening shortness of breath and malaise. He had no significant past medical history and denied any alcohol, tobacco, or recreational drug use. His triage vital signs were temperature 39.5^o^C, heart rate 117 beats per minute, blood pressure of 122/67 mm Hg, respiratory rate of 30 breaths per minute, and pulse oximetry of 99% on room air. Significant laboratory results were as follows: white blood cell count 16.4 K/mm3; C-reactive protein 129.3 mg/L; ferritin 333 ng/mL (reference range 22.0–275 ng/mL); and procalcitonin 4.76 ng/mL (0.15 ng/mL). His initial PE was significant for a new IV/VI grade systolic ejection murmur hear at the base of the heart.

Initial blood cultures were obtained in the ED. He had negative screening tests for both severe acute respiratory syndrome coronavirus 2 and human immunodeficiency virus. A point-of-care TTE in the ED showed severe mitral regurgitation and concern for endocarditis. The patient was started on IV vancomycin and piperacillin-tazobactam. On hospital day two, admission blood cultures grew aerobic Gram-negative diplococci, and the patient was transitioned to ceftriaxone and ciprofloxacin. The organisms identified were *N. gonorrhoeae,* again using the VITEK analyzer. The isolate was sensitive to ceftriaxone. Further urine testing discovered only chlamydia, which was treated with azithromycin. The patient underwent mitral valve repair on hospital day 10, and he completed six weeks of IV ceftriaxone.

## DISCUSSION

This case series of three separate cases of DGI with development of endocarditis within a three-year period at a single institution has not been reported previously. Evidence suggests that asymptomatic infection may increase the risk of DGI, as it may lead to delay in diagnosis and subsequent antibiotic treatment. Thus, a lack of genital symptoms should not remove this from the differential.^10^

This case series shows some important atypical features that should serve to help increase awareness on the varied ways that DGI can present in the ED. Previous literature describes an increased prevalence of DGI and endocarditis in males^1^; however, our case series contains two women and one man. This series displays the insidious nature of this disease: one patient, despite multiple blood cultures obtained, was never able to culture the organism, yet she displayed a classic migratory rash that can accompany DGI and developed endocarditis on the aortic valve. Another patient was able to have the organism identified directly from blood cultures but had negative cervical swabs for infection and a history of previous chlamydia infection. Lastly, the male patient had no prior recorded sexually transmitted infection and no reported genital symptoms. He ultimately presented to the ED for his “initial” infection with fulminant endocarditis with positive blood cultures, but his urine was positive for chlamydia alone.

Several factors may be contributing to an overall increase in DGI and endocarditis. *N. gonorrhoeae* has been increasing in incidence across all races/ethnicities and is currently the second most commonly reported sexually transmitted infection in the US.^6^ Along with this overall increasing incidence, there has also been emergence of cephalosporin-resistant strains, which has prompted the US Centers for Disease Control and Prevention to update recommendations for treatment, including an increase in the dose of intramuscular ceftriaxone from 250 mg to 500 mg. Patients weighing more than 150 kilograms should be given 1 g of ceftriaxone.^7^–^9^

Evaluation in the ED for sexually transmitted infections should thus include a full skin and joint exam and a full cardiac exam. There should also be a low threshold to obtain an echocardiogram if any abnormalities are discerned. Overall, emergency physicians should consider DGI in all patients presenting with oligoarthralgia, polyarthralgia, flu-like symptoms, dermatitis, tenosynovitis and, as noted in this case series, endocarditis. The constellation of arthritis and dermatitis with or without tenosynovitis may occur 2–3 weeks after the primary infection. Vesicular and/or pustular lesions are often painless; so a thorough physical examination of their common sites, the distal upper and lower extremities, is warranted. Tenosynovitis classically involves multiple tendons, also of the distal upper and lower extremities. Arthritis is more often migratory in nature and asymmetric.

## CONCLUSION

This case series highlights three divergent and atypical cases at one hospital system over a recent three-year period. The insidious nature of disseminated gonoccal infection and the atypical presentation of endocarditis in two women and one man within this series should serve as a warning sign for all emergency physicians to become more aware of this dangerous and deadly complication of possible untreated, or under-treated, gonorrhea. Increased surveillance for *N. gonorrhoeae* should be implemented across the US with rapid and widespread notification of all new cases of DGI/endocarditis to alert emergency physicians to either overall increasing incidence or penetrance of antibiotic-resistant strains.

The authors attest that their institution requires neither Institutional Review Board approval nor patient consent for publication of this case series. Documentation on file.

## Figures and Tables

**Image:**
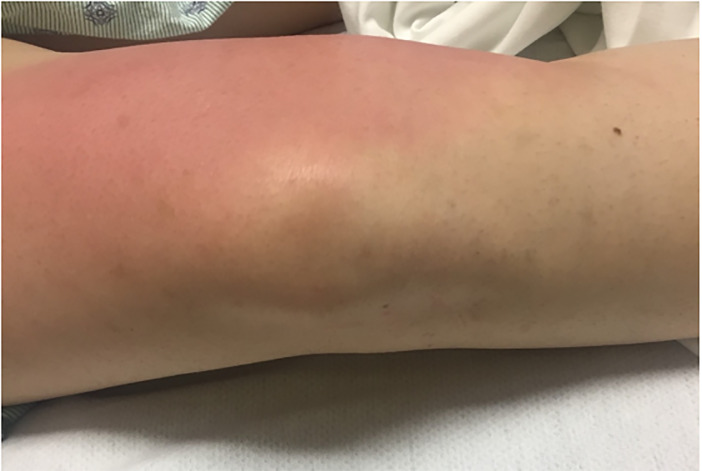
Erythematous rash of right knee.
